# Title: P2x7 Receptor Activation and Estrogen Status Drive Neuroinflammatory Mechanisms in a Rat Model for Dry Eye

**DOI:** 10.3389/fphar.2022.827244

**Published:** 2022-04-05

**Authors:** David A. Bereiter, Mostafeezur Rahman, Fabeeha Ahmed, Randall Thompson, Nhungoc Luong, Julie K. Olson

**Affiliations:** Department of Diagnostic and Biological Sciences, School of Dentistry, University of Minnesota, Minneapolis, MN, United States

**Keywords:** dry eye, microglia, estrogen (17b-estradiol), trigeminal afferent pathway, purinergic (P2X) receptors

## Abstract

Dry eye disease (DED) is recognized as a chronic inflammatory condition with an increase in tear osmolarity and loss of tear film integrity. DED is often accompanied by adverse ocular symptoms which are more prevalent in females than males. The basis for ocular hyperalgesia in DED remains uncertain; however, both peripheral and central neural mechanisms are implicated. A model for aqueous deficient DED, exorbital gland excision, was used to determine if activation of the purinergic receptor subtype 7, P2X7R, expressed by non-neural cells in peripheral and central trigeminal nerve pathways, contributed to persistent ocular hyperalgesia. Densitometry of trigeminal brainstem sections revealed increases in P2X7R, the myeloid cell marker Iba1, and the inflammasome, NLRP3, of estradiol-treated DED females compared to estradiol-treated sham females, while expression in DED males and DED females not given estradiol displayed minor changes. No evidence of immune cell infiltration into the trigeminal brainstem was seen in DED rats; however, markers for microglia activation (Iba1) were increased in all groups. Isolated microglia expressed increased levels of P2X7R and P2X4R, IL-1*β* (Ιnterleukin-1*β*), NLRP3, and iNOS (nitric oxide synthase). Further, estradiol-treated DED females displayed greater increases in P2X7R, IL-1*β* and NLRP3 expression compared to untreated DED females. Orbicularis oculi muscle activity (OOemg) evoked by ocular instillation of hypertonic saline (HS) was recorded as a surrogate measure of ocular hyperalgesia and was markedly enhanced in all DED groups compared to sham rats. Systemic minocycline reduced HS-evoked OOemg in all DED groups compared to sham rats. Local microinjection in the caudal trigeminal brainstem of an antagonist for P2X7R (A804598) greatly reduced HS-evoked OOemg activity in all DE groups, while responses in sham groups were not affected. Intra-trigeminal ganglion injection of siRNA for P2X7R significantly reduced HS-evoked OOemg activity in all DED groups, while evoked responses in sham animals were not affected. These results indicated that activation of P2X7R at central and peripheral sites in trigeminal pain pathways contributed to an increase in ocular hyperalgesia and microglia activation in DED males and females. Estrogen treatment in females further amplified ocular hyperalgesia and neuroimmune responses in this model for aqueous deficient DED.

## Introduction

Dry eye disease (DED) is a chronic inflammatory condition that is influenced by multiple intrinsic and external factors (Craig et al., 2017; Pflugfelder and Paiva 2017). Persistent adverse symptoms are the main reasons patients seek medical attention for DED (Rosenthal et al., 2009; Galor et al., 2015) which can range from a sense of ocular dryness to severe pain (Begley et al., 2001; Kalangara et al., 2017). Management of ocular symptoms in moderate to severe cases of DED is often inadequate (Asbell and Spiegel 2010; Williamson et al., 2014; Siedlecki et al., 2020). Although considerable progress has been made in the diagnosis of DED (Wolffsohn et al., 2017), the neural mechanisms that mediate ocular hyperalgesia are not well defined. It is widely accepted that most chronic pain conditions involve both peripheral and central neural mechanisms (Baron et al., 2013; Grace et al., 2021); however, studies concerned with the mechanisms of adverse symptoms in DED have emphasized peripheral factors (Belmonte et al., 2017). Peripheral biomarkers alone are not sufficient to predict the intensity of adverse ocular symptoms (Bron et al., 2014; Sullivan 2014) suggesting that CNS as well as peripheral neural mechanisms are involved.

Neuroimmune interactions are critical for the maintenance of inflammatory and neuropathic pain (Ji et al., 2016; Hore and Denk 2019). Tear hyperosmolarity is a prominent diagnostic feature of DED which is thought to trigger ocular infiltration of immune cells in a “vicious cycle” resulting in chronic inflammation (Baudouin et al., 2013). Tears of DED patients contain elevated levels of pro-inflammatory cytokines and adenosine triphosphate (ATP) (Guzman-Aranguez et al., 2017; Willcox et al., 2017). ATP released from injured cells enhances inflammation and immune responses through activation of the purinergic receptors such as P2X7R (Burnstock 2016; Di Virgilio et al., 2017). Disruption of P2X7R markedly reduces behavioral correlates of inflammatory and neuropathic pain in animals (Chessell et al., 2005), while mutations of the P2X7R gene significantly influence pain intensity in humans (Sorge et al., 2012; Kambur et al., 2018). P2X7R is highly expressed by several immune cell types, while expression by neurons remains controversial (Kaczmarek-Hajek et al., 2018). In the trigeminal sensory system, P2X7R is expressed by satellite glia which surround trigeminal ganglion (TG) neurons (Nowodworska et al., 2017; Inoue and Tsuda, 2021) and by microglia in the trigeminal brainstem (Ito et al., 2013). The threshold concentration of ATP necessary for P2X7R activation is higher than for other ionotropic purinergic receptors consistent with a role during moderate to severe inflammatory conditions. P2X7R activation is critical for assembly of the inflammasome, NLRP3, which through caspase-1 activation is necessary for IL-1*β* production and release by glia and immune cells (Burnstock 2016; Di Virgilio et al., 2017).

Women are diagnosed with DED more often and display more severe ocular symptoms than men (Sullivan et al., 2017; Vehof et al., 2018); however, the basis for sex differences in symptomatic DED remains unresolved. In an animal model for aqueous deficient DED, female mice displayed greater nociceptive and anxiety-like behaviors than males (Mecum et al., 2020). Estrogen status has long been recognized as a contributing factor for sex differences in pain behavior (Amandusson and Blomqvist 2013). While sex differences in microglia activation have been reported (Guneykaya et al., 2018), the relative contributions of activated microglia in male and female animals to pain processing are not well defined (Sorge et al., 2015; Taves et al., 2015; Lopes et al., 2017; Fernandez-Zafra et al., 2019). The present study tested the hypothesis that elevated estrogen status in ovariectomized (OvXE) female rats is a significant factor in mediating microglia activation and evoked orbicularis oculi muscle activity (OOemg) in a model for aqueous deficient DED. These results demonstrated that activation of P2X7R at peripheral and central sites in trigeminal pain pathways played a significant role in mediating neuroimmune responses and ocular hyperalgesia in DED.

## Materials and Methods

***Animals***. A total of 288 adult male, ovariectomized female (OvX) and estradiol-treated OvX female (OvXE) rats (250–350 g, Sprague-Dawley, Harlan, Indianapolis, IN) were used in these experiments. OvXE rats were given a single bolus injection of estradiol (E2, 30 μg/kg, sc) the day before tissue collection or muscle recording to simulate the proestrus surge in estrogens in normal cycling rats (Naftolin et al., 1972). The estrogen status of female rats was determined on the day of the experiment by vaginal cytology. Vaginal lavage samples from OvX rats contained small nucleated leukocytes, while samples from OvXE rats displayed mainly large nucleated epithelial cells consistent with low and high estrogen conditions, respectively. Animals were housed in pairs and given access to food and water ad libitum. Climate and lighting were controlled (25 ± 2°C, 12:12-h light/dark cycle, light on at 7:00 a.m.). The animal protocols were approved by the Institutional Animal Care and Use Committee of the University of Minnesota (United States) and according to guidelines set by The National Institutes of Health Guide for the Care and the Use of Laboratory Animals (PHS Law 99-158, revised 2015).

***Exorbital gland excision*.** Rats were anesthetized with isoflurane (5%) and the overlying masseter muscle was exposed. A small skin incision was made over the masseter muscle to remove the left exorbital gland. The wound margin was treated with 2% xylocaine gel and the incision was closed with absorbable sutures. The gland was exposed in sham rats but was not removed. Carprofen (25 mg/kg, i.p) was given as a single dose after surgery. Rats survived for 2 or 14 days after gland removal for experiments that involved tissue collection and cellular and molecular analyses. Orbicularis oculi muscle recording was preformed 14 days after surgery.

### Tear Volume and Orbicularis Oculi Muscle Electromyography (OOemg) Recording.

Rats were anesthetized with urethane (1.2–1.5 g/kg, ip) and spontaneous tear volume was measured by the increase in wet length of phenol red thread (ZONE-QUICK, Menicon INC., San Mateo, CA) at 14 days after surgery. The thread was gently placed in contact with the cornea/conjunctiva at its inferior-lateral edge and tear volume was measured over 2 min. Following tear volume measurement, a cannula was positioned in the left femoral artery to monitor mean arterial blood pressure and was maintained at 90–110 mmHg. Wound margins were infiltrated with 2% lidocaine and body temperature was kept at 38°C with a heating blanket. Rats were allowed to breathe spontaneously. The rat was placed in a stereotaxic frame and Teflon-coated copper wires (0.12 mm diameter) were implanted by a 26-gauge needle near the center of the upper and lower OO muscles, proximal to the lid margins, and grounded by a wire inserted in the neck muscle and at least 1 h elapsed before recording began (Rahman et al., 2017).

OOemg activity was sampled at 1,000 Hz, amplified (x10 k), filtered (bandwidth 10–300 Hz), displayed and stored offline (ADInstruments, Colorado spring, CO, United States). OOemg activity was recorded continuously for 6 min from 3 min before (baseline activity) until 3 min after stimulus onset. Recorded activity was rectified and stored as 1 s bins for off-line analyses. Total OOemg activity was calculated initially from the raw signal and the integrated area under the curve (AUC) for the 3 min epoch (μV-s per 3 min) sampled after each stimulus minus the 3 min epoch recorded immediately prior to stimulation. Ocular surface stimulation consisted of instillation of normal saline (0.15M NaCl) and hypertonic saline (HS) in concentrations of 1 and 2.5 M NaCl. Previously we determined that HS-evoked total OOemg activity consisted mainly (>90% of total) of a period of long duration activity (>200 ms) and a minor contribution from short duration (<200 ms) and that total OOemg activity was a valid measure of ocular hyperalgesia in a rat model for DED (Rahman et al., 2017; Bereiter et al., 2018).

### Effects of Drug Treatments on OOemg Activity

*OOemg and systemic minocycline*. The non-specific anti-inflammatory agent and glial cell inhibitor, minocycline, was given systemically (40 mg/kg, ip) for 4 days prior to recording (four to five rats per group). At 14d after gland removal (4 days after the onset of minocycline treatment) rats were prepared for OOemg recording as detailed above. OOemg activity was evoked by ocular instillation of normal saline (0.15M) followed by increasing concentrations of HS (1.0, 2.5 M) applied at 30 min intervals. Each solution remained on the eye for 3–4 min before rinsing with artificial tears. Although the highest osmolar concentrations of HS used in this study was greater than that reported to evoke pain sensation in humans (Liu et al., 2009) or squint-like behavior in conscious rats (Yorek et al., 2016) (600–1,000 mOsm (by sucrose) versus 900-2200mOsM (by NaCl), respectively), we found that brief repeated application of these higher HS concentrations did not induce desensitization or tachyphylaxis of evoked OOemg activity.

*OOemg and local inhibition of purinergic P2X7R in trigeminal brainstem*. Rats were prepared for OOemg recording (10 rats per group) as noted above. Next the dorsal surface of the caudal brainstem was exposed surgically to allow microinjection of the selective P2X7 receptor antagonist, A804598 (10 μM, 0.2µL, Tocris) into trigeminal subnucleus caudalis (Vc) ipsilateral to the ocular stimulus. OOemg activity was evoked by ocular instillation of 2.5 M NaCl before drug injection. After 20 min A804598 or vehicle (PBS) was injected into Vc and 2.5M NaCl was applied to the ocular surface at 10, 30 and 50 min after drug injection. Drugs were prepared fresh each day. In separate animals, A804598 (10 mg/kg, sc) was given daily for 4 days and then the trigeminal brainstem was removed to determine the effects of drug treatment on markers for microglia activation in sham and 14 days DED rats.

*OOemg after intra-trigeminal ganglion (TG) injection of siRNA for P2X7R.* Rats were anesthetized with pentobarbital sodium (50 mg/kg, i.p) and maintained with isoflurane (1–2%). The animals were placed in a stereotaxic apparatus, the scalp was exposed, and a hole was drilled into the left parietal bone (3.5–4 mm anterior to the auricle and 3–4 mm lateral to the midline, and 8 mm below the cortical surface). The siRNA solution (600 μg, 200 nL, Stealth RNAi for P2X7R, RSS-310828 #83546611, validated by Invitrogen, Carlsbad, CA) or the Stealth RNAi negative control (#12935112, lot 857,979, Invitrogen), was injected into the left TG ∼10 days after exorbital gland removal via a 33-gauge needle inserted through a 26-gauge guided cannula positioned stereotaxically and was kept in position at least 10 min after the injection to minimize leakage. The wound margin was closed with sutures and povidone-iodine solution was applied to the wound area. A single dose of carprofen (25 mg/kg, i.p) was injected in each animal to minimize post-surgical pain. Animals survived for 3 days after the intra-trigeminal ganglion injection (i.e., 10 days after gland removal). OOemg activity was evoked by ocular instillation of normal saline (0.15M) followed by increasing concentrations of HS (1.0, 2.5 M) applied at 30 min intervals. Each solution remained on the eye for 3–4 min before rinsing with artificial tears. At end of the recording session the TG and spinal trigeminal brainstem (Vsp) were removed and prepared for protein analyses by western blot. P2X7R protein levels were measured in TG samples to confirm transcription knockdown, while Iba1 was measured in Vsp samples to assess the effects on microglia actvation.

***Immunohistochemistry.*** Male, OvX and OvXE rats (sham, 2 days, 14 days-post surgery, four rats per group) were anesthetized with pentobarbital (70 mg/kg) and then perfused with phosphate buffered saline (PBS) followed by 4% paraformaldehyde (PFA). The caudal brainstem was removed and placed in PFA overnight at 4°C. Transverse tissue sections were cut at 30µm on a vibratome and free-floating sections were blocked for 1 h (PBS, 0.1% Triton X-100, 1% donkey serum) and then incubated overnight at 4°C with primary antibodies (Ab) at 1:1,000 dilution for microglia (Iba-1, MABN92, Millipore), P2X7R (APR004, Alomone), NLRP3 (orb101128, Biorbyt) and GFAP (ABnova MAB10760, Walnut, CA, 1:500). Sections were washed in PBS (3 × 5 min) and incubated in secondary Ab (donkey antirabbit IgG biotin, AP182B, Millipore) for 90 min. Staining was visualized by Vector ABC compound (ABC kit, PK-4000, peroxidase standard) for 1 h at room temperature and color developed with diaminobenzidine tetrahydrochloride (peroxidase substrate, SK-4100) for 90 s. Sections were washed, air dried and mounted. Stained sections from sham and 14d DED rats were analyzed by light microscopy (Olympus BX51) at 10X magnification and quantified by densitometry (4 sections/rat) using ImageJ software. Controls for immunohistochemistry were processed by incubating sections without primary antibodies. Sections were analyzed ipsilateral to exorbital gland removal without prior knowledge of treatment. ***Immunofluorescence.*** Briefly, representative examples of trigeminal brainstem tissues were paraffin embedded and cut at 20 µm on a microtome. Sections were dewaxed, hydrated and stained with primary antibodies at a 1: 300 dilution: anti- P2X7 Cell Signaling # 13,809, Danvers, MA; anti-Iba-1, Millipore #MABN92, Temecula, CA; anti- NLRP3/Cryopyrin, Biorbyt #101128, St. Louis, MO). Sections were rinsed and incubated with appropriate secondary antibodies at 1:500 dilution: anti rabbit CY5 or anti-mouse CY2, Jackson Immunoresearch #s 711-175-152 and 715-226-151, West Grove, PA. Images were captured using a Zeiss LSM700 confocal microscope with 40 × objective.

***Western Blot.*** Aliquots of protein (20 µg) were run on 4–20% polyacrylamide gels, transferred to 0.45 µm membranes, and incubated with anti- P2X7R antibody (1-1,000 dilution, Cell Signaling, #13809, Danvers, MA. Anti- GAPDH was used as normalizing antibody (1, 1:000 dilution, Sigma Chemical, # WH0002597M1, St. Louis MO). Secondary antibodies were IRDye 800CW anti-mouse and IRDye 680RD anti-rabbit, 1-15,000 dilution, LICOR, Lincoln NE. Membranes were scanned on LICOR Odyssey infrared scanner.

***Flow cytometry.*** Rats were anesthetized and perfused through the heart with PBS, and the spinal trigeminal brainstem (Vsp), which included subnucleus oralis (Vo), interpolaris (Vi) and caudalis (Vc) regions, was dissected (3 rats per group). The tissue samples were minced and digested with collagenase type IV (Invitrogen) and DNAse (Invitrogen) for 30 min at 37°C. The tissue was then dissociated through nylon mesh before the mononuclear cells were separated on a 70/30 percoll gradient. The mononuclear cells were washed with FACS buffer (PBS with 5% normal goat serum) and blocked with antibody to CD16/32 (BD Bioscience) at 4°C for 30 min and then incubated for 45 min at 4°C with fluorescently labeled antibodies specific for CD45, CD11b, CD8, and CD4. The cells were analyzed on a FACScalibur (BD Bioscience) based on live cells. The CD45 intermediate CD11b^+^ cells were resident microglia, and the CD45 high cells were analyzed to determine the number of macrophage (CD11b^+^), CD4^+^ T cells, and CD8^+^ T cells.

***Cell sorting.*** Rats were perfused with PBS, and the Vsp, which included Vo, Vi and Vc subnuclei, was dissected. The brainstems were used in Neural Tissue Dissociation Kit (P) following the protocol. The resulting cells were separated on a 70/30 percoll gradient. The mononuclear cells were incubated with CD11b^+^ microglia MicroBeads (Miltenyi) and separated on a column to obtain a specific population of microglia. The isolated microglia were >99% pure based on flow cytometric analysis.

***RNA isolation and quantitative real time PCR*** (***qRT-PCR***) ***.*** RNA was isolated from microglia using SV Total RNA Isolation kit which contains a DNAse reaction (Promega). First strand cDNA was generated from 1 μg of total RNA using oligo (dT)_12-18_ primers and Advantage for RT-PCR kit in a final volume of 100 μL (Clontech). qRT-PCR was conducted in triplicate with Rotor-Gene SYBR green RT-PCR kit (Qiagen). Briefly, 0.5μM primers, 1X SYBR Green reagent, and 2μL of cDNA were combined in 10μL reactions. The primers were specific for *β*-actin, IL-1*β*, TNF*α*, NLRP3, iNOS, BDNF, and TLR4. qRT-PCR was conducted on a Rotor-Gene Qiagen Q instrument using hot start with cycle combinations, 40 cycles: 95°C for 15s; 60°C for 20s; 72°C for 15s, followed by a melt from 75 to 95°C. Quantitation of the mRNA was based on standard curves derived from cDNA standards for each primer pair. Specific mRNA expression was normalized to *β*-actin expression.

### Experimental Design and Statistical Analysis

**FIGURE 1:**
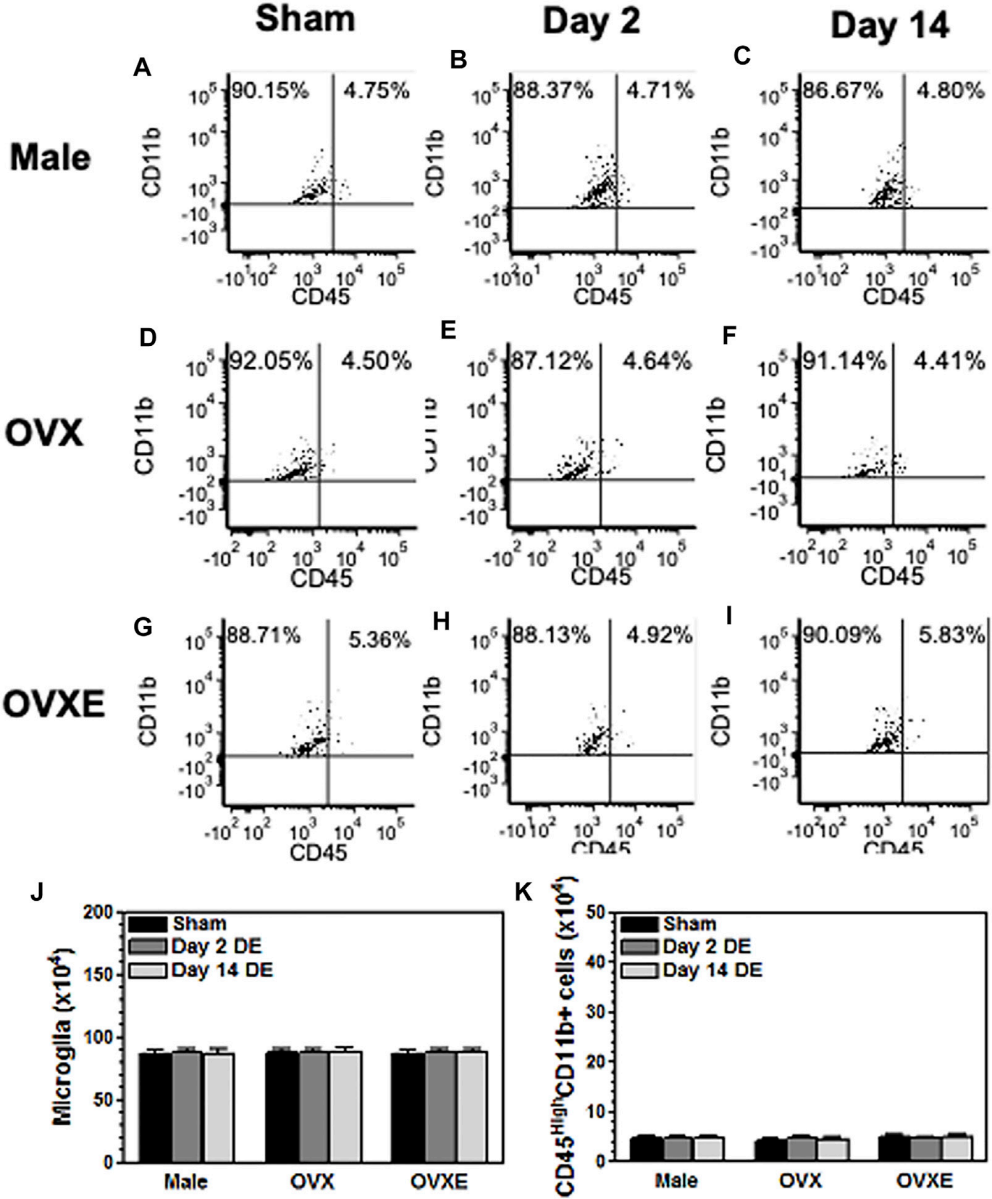
Peripheral immune cells do not infiltrate the brainstem during DED. Male, OVX, and OVXE rats had the left exorbital gland removed or were sham treated **(A,D,G)** (3 rats per group). At 2 days **(B,E,H)** and 14 days **(C,F,I)** post-surgery, rats were perfused with PBS and brainstem tissue was dissociated with trypsin and collagenase prior to separation on a 70/30 percoll gradient. The mononuclear cells were incubated with fluorescently labeled antibody for CD45 and CD11b. The cells were analyzed by flow cytometry gating on live cells. The infiltrating monocytes/macrophage identified as CD45highCD11b^+^ and microglia identified as CD45intermediateCD11b^+^. The percentage in each quadrant is based on total mononuclear cells isolated from the brainstem. These dot plots represent data from one of three independent repeated experiments. The total number of microglia (CD45intermediateCD11b+) **(J)** and monocytes/macrophage (CD45HighCD11b^+^) **(K)** were calculated for each of the three experiments and combined in the graphs **(J,K)**. *N* = 3 rats per group.

**FIGURE 2:**
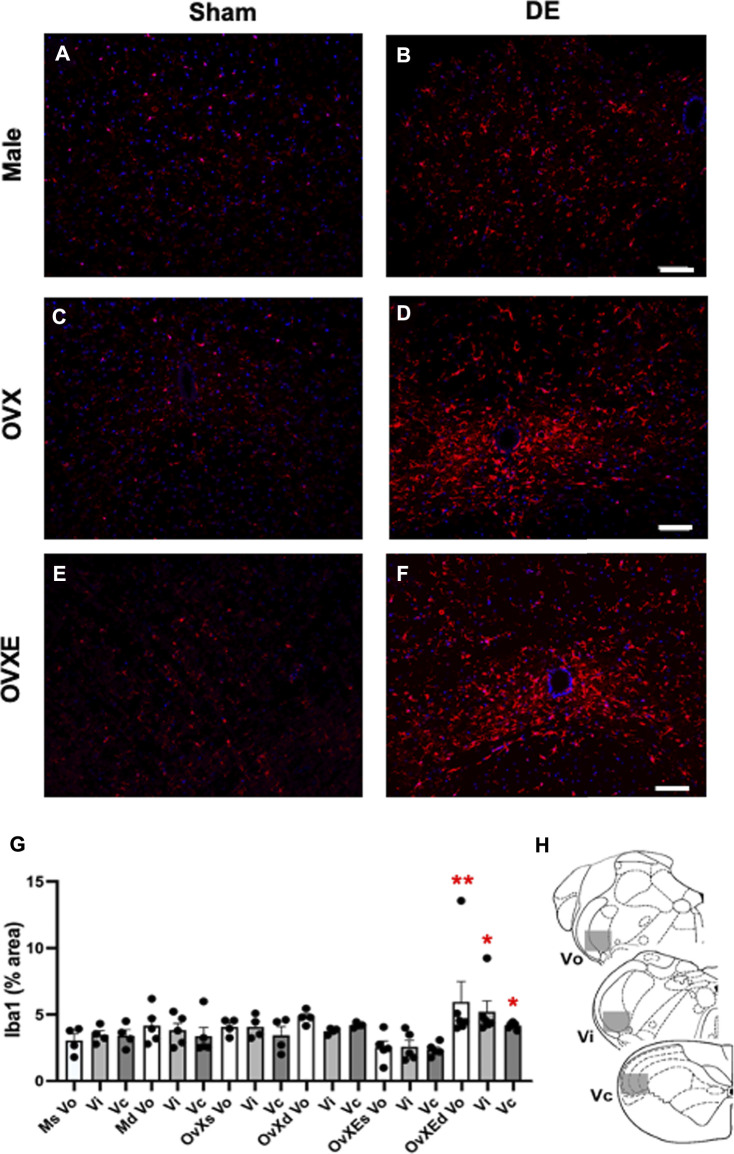
Microglia are activated in the Vsp during DED and express Iba-1. Male, OVX, and OVXE 14 days DED rats **(B,D,F)** and sham rats **(A,C,E)** were perfused with 4% PFA, fixed and brainstem sections stained with anti-Iba1 (red) and dapi (blue). The micrographic examples are of trigeminal subnucleus caudalis (Vc). Scale bar = 80 µm. **(G)** Densitometry was conducted on light microscopy-stained sections from Vo, Vi, Vc regions using ImageJ on four sections per rat with four rats/group (mean ± SEM). Abbreviations: Ms, male sham; Md, male DED; OvXs, OvX sham; OvXd, OvX DED; OvXEs, OvXE sham; OvXEd, OvXE DED. *p < 0.05, **p < 0.01 versus sham group. **(H)** Shaded areas represent regions of trigeminal brainstem that were sampled. *N* = 4 rats per group.

**FIGURE 3:**
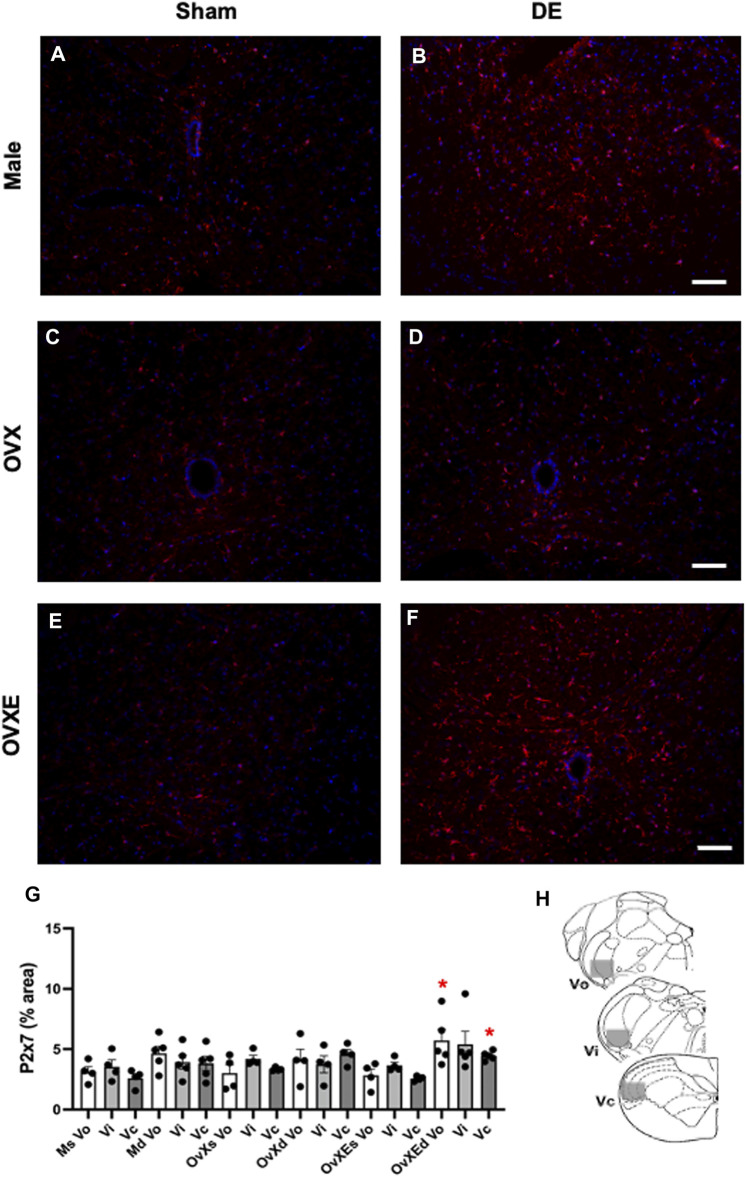
Microglia express P2X7R in the Vsp during DED. Male, OVX, and OVXE 14 days DED rats **(B,D,F)** and sham rats **(A,C,E)** were perfused with 4% PFA, fixed and brainstem sections stained with anti-Iba1 (red) and dapi (blue). The micrographic examples are of trigeminal subnucleus caudalis (Vc). Scale bar = 80 µm. **(G)** Densitometry was conducted on Vo, Vi, Vc regions using ImageJ (mean ± SEM). Abbreviations: Ms, male sham; Md, male DED; OvXs, OvX sham; OvXd, OvX DED; OvXEs, OvXE sham; OvXEd, OvXE DED. *p < 0.05 versus sham group. **(H)** Shaded areas represent regions of trigeminal brainstem that were sampled. *N* = 4 sections per rat and four rats/group.

**FIGURE 4:**
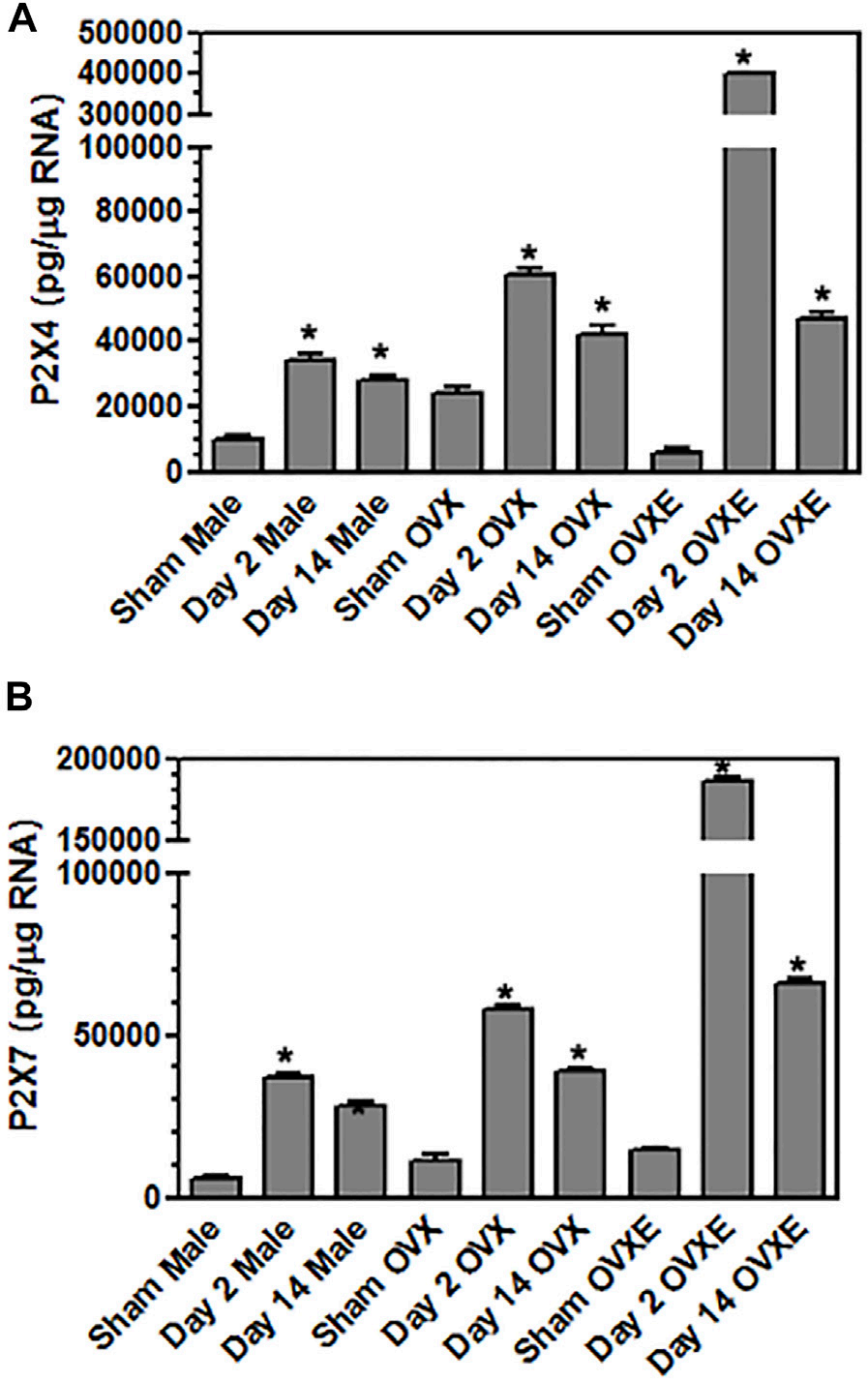
Microglia express purinergic receptors in an estrogen dependent manner during DED. Male, OVX, and OVXE rats had the left exorbital gland removed or were sham treated and at 2 and 14 days post-surgery, rats were perfused with PBS, trigeminal brainstem was removed and tissue dissociated prior to separation on a 70/30 percoll gradient. The mononuclear cells were incubated with fluorescently labeled antibody for CD45 and CD11b. The microglia were sorted on a cell sorter based on CD45intermediateCD11b^+^ cells. The isolated microglia were lysed, RNA isolated, converted to cDNA, and analyzed by real time PCR for expression of **(A)** P2X4, **(B)** P2X7. Significant differences were determined by one-way ANOVA and Bonferroni’s multiple comparison test (*p < 0.001) based on sham rat microglia for the corresponding group (mean ± SEM). *N* = 8 rats per group. Data presented as mean ± SEM.

**FIGURE 5:**
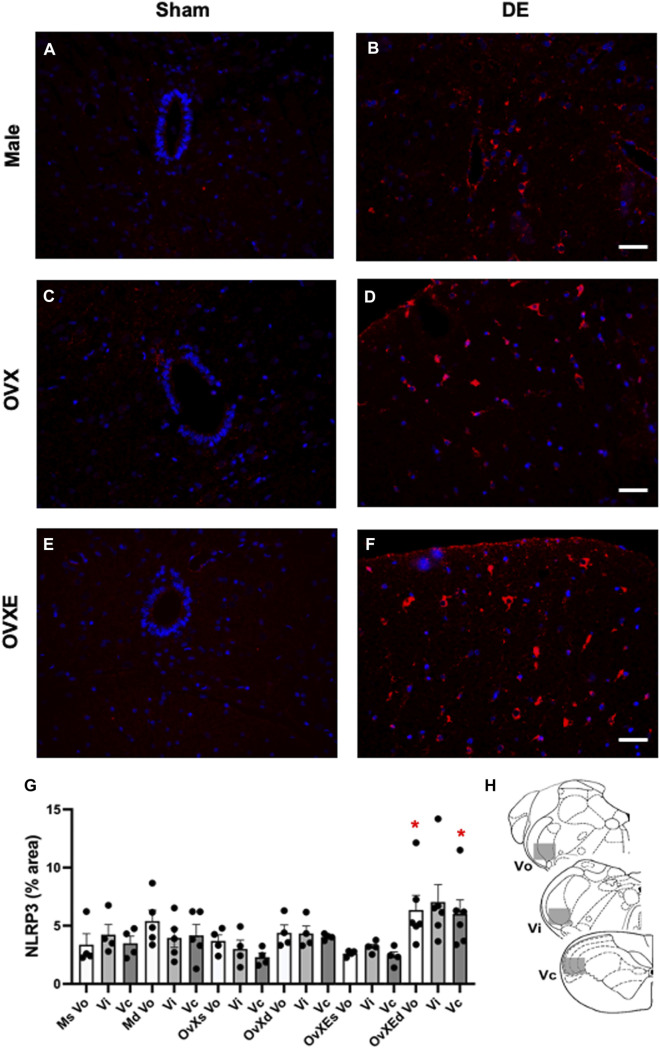
Microglia have increased NLRP3 expression in the brainstem during DED. Male, OVX, and OVXE 14 days DED rats **(B,D,F)** and sham rats **(A,C,E)** were perfused with 4% PFA, fixed and brainstem sections stained with anti-Iba1 (red) and dapi (blue). The micrographic examples are of trigeminal subnucleus caudalis (Vc). Scale bar = 80 µm. **(G)** Densitometry was conducted on light microscopy-stained sections at Vo, Vi, Vc regions using ImageJ on four sections per rat with four rats/group (mean ± SEM) Abbreviations: Ms, male sham; Md, male DED; OvXs, OvX sham; OvXd, OvX DED; OvXEs, OvXE sham; OvXEd, OvXE DED. *p < 0.05 versus sham group. **(H)** Shaded areas represent regions of trigeminal brainstem that were sampled. *N* = 4 rats per group.

**FIGURE 6:**
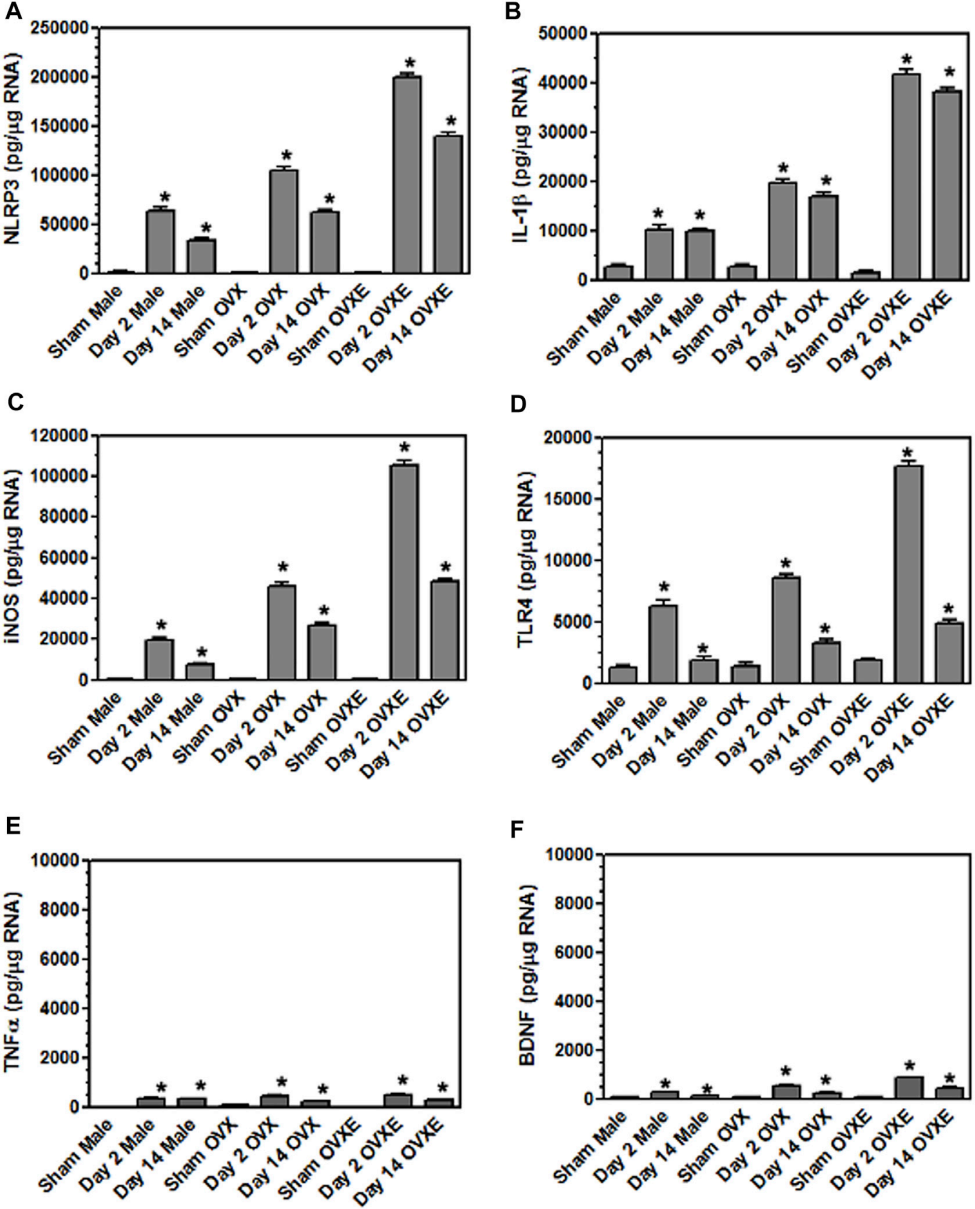
Microglia express inflammatory cytokines and effector molecules in an estrogen dependent manner during DED. Male, OVX, and OVXE rats had their exorbital glands removed or sham treated. At 2 days and 14 days post-surgery, brainstem tissue was prepared and analyzed by real time PCR as in Figure 4 for expression of: **(A)** IL-1*β*, **(B)** NLRP3, **(C)** iNOS, **(D)** TLR4, **(E)** TNF*α*, and **(F)** BDNF. *p* < 0.001 versus sham group. *N* = 8 rats per group.

**FIGURE 7:**
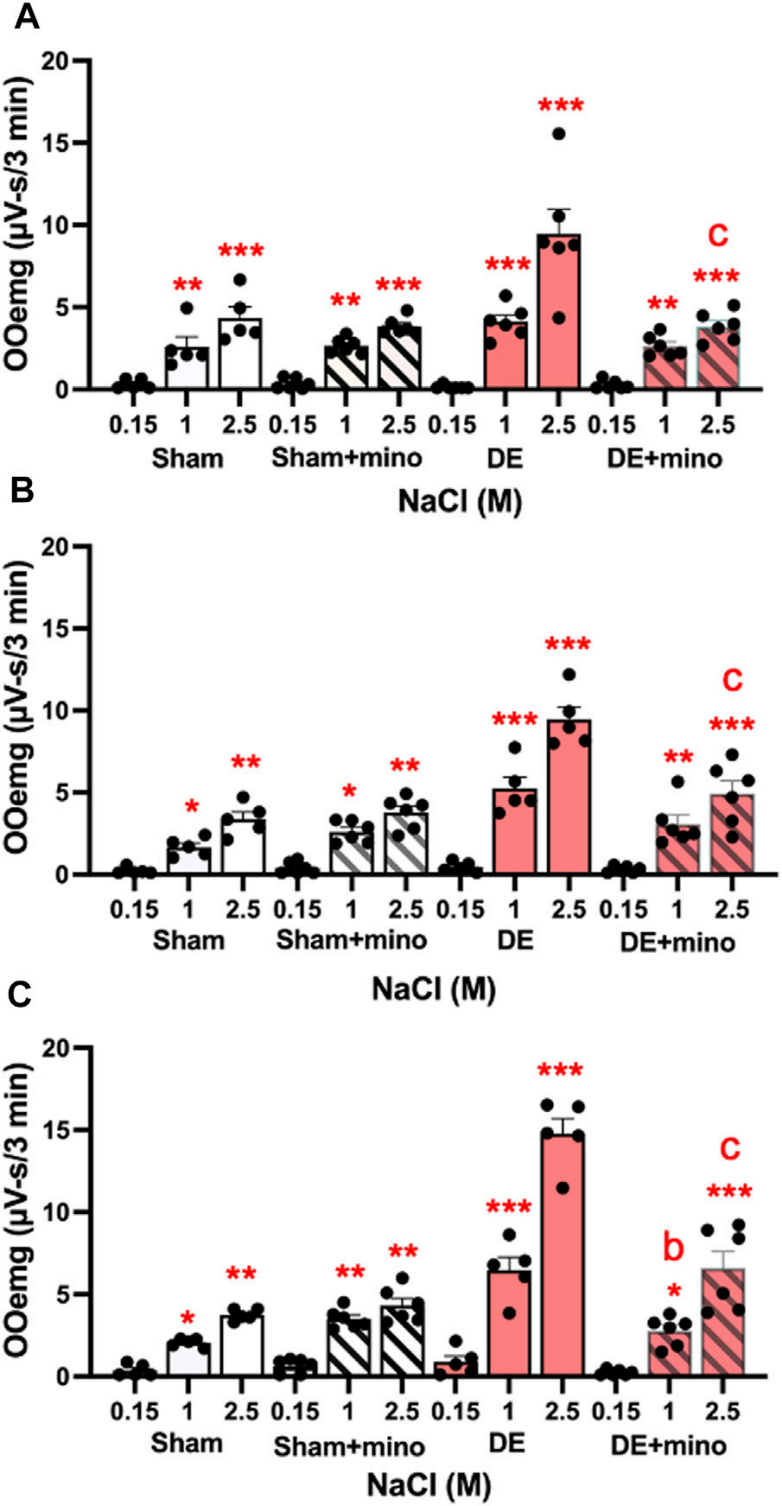
Minocycline reduces HS-evoked OOemg activity in DED males and females. Minocycline (40 mg/kg/day, sc, X 4 days) significantly reduced the enhanced HS-evoked OOemg activity recorded 14 days after exorbital gland removal. Test stimuli = ocular instillation of NaCl (0.15, 1.0, 2.5 M). Test stimuli applied at 30 min intervals. **(A)** Males; **(B)** OvX; **(C)** OvXE. Open bars = sham; open shaded bars = sham + minocycline; open red bars = 14 days DED; red shaded bars = 14 days DED + minocycline. *p < 0.05, **p < 0.01, ***p < 0.001 versus 0.15 M NaCl; b = *p*< 0.01; c = *p*< 0.001 versus pre-drug. Sham, *n* = 5-6 rats per group; DED, *n* = 6 per group. Data presented as mean ± SEM.

**FIGURE 8:**
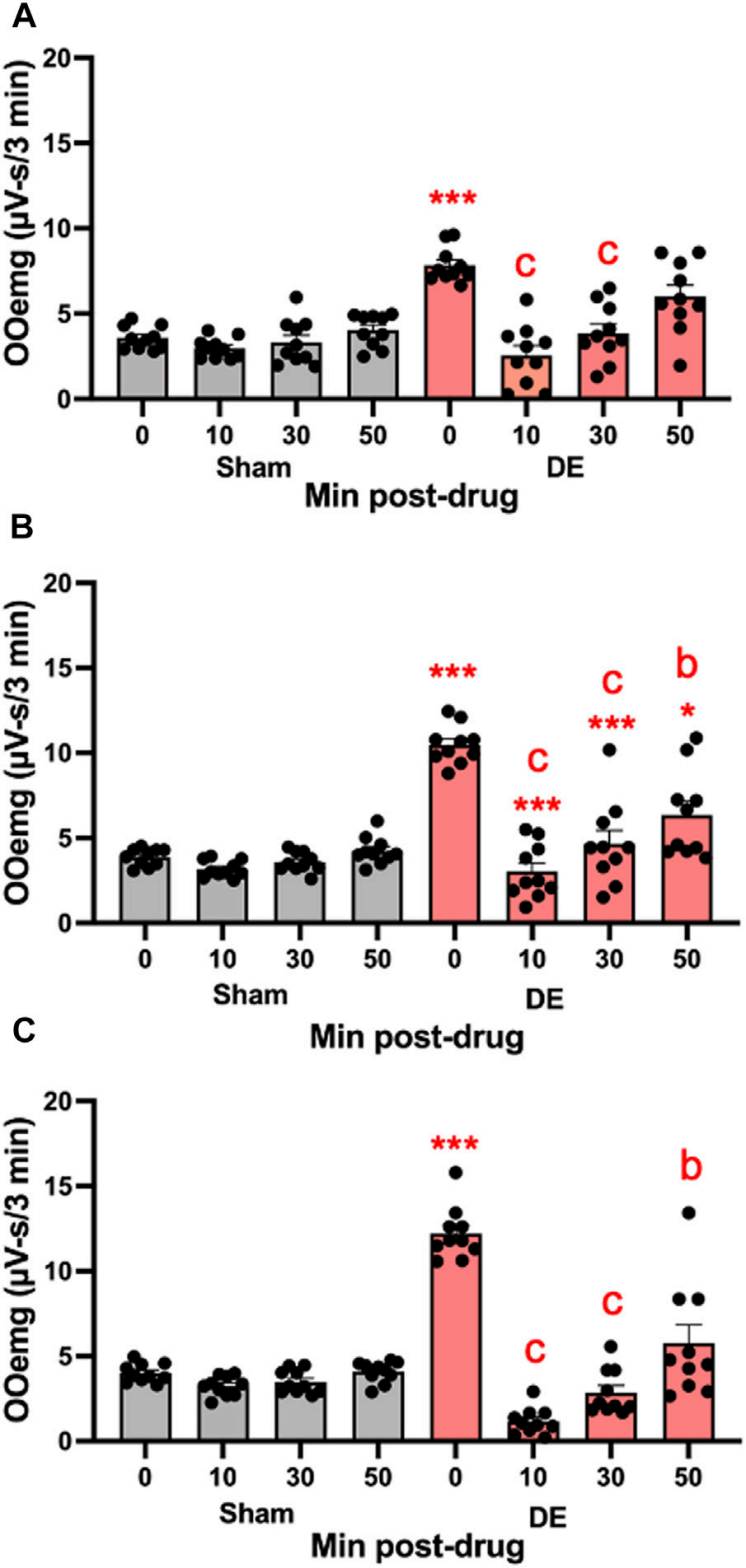
Local inhibition of P2X7R in Vc reduces HS-evoked OOemg activity during DED. The selective P2X7R antagonist (A804598) was microinjected (10 μM, 0.2 µL) into the Vc of sham and 14 days DED rats 30 min prior to the test stimulus (ocular instillation of 2.5 M NaCl). **(A)** Males, **(B)** OvX females, **(C)** OvXE. Gray bars = sham; red bars = 14-day DED. *p < 0.01, *p* < 0.001 versus 0.15 M NaCl; *b* = *p*< 0.01, *c* = *p*< 0.001 versus pre-drug. *N* = 10 rats per group.

**FIGURE 9:**
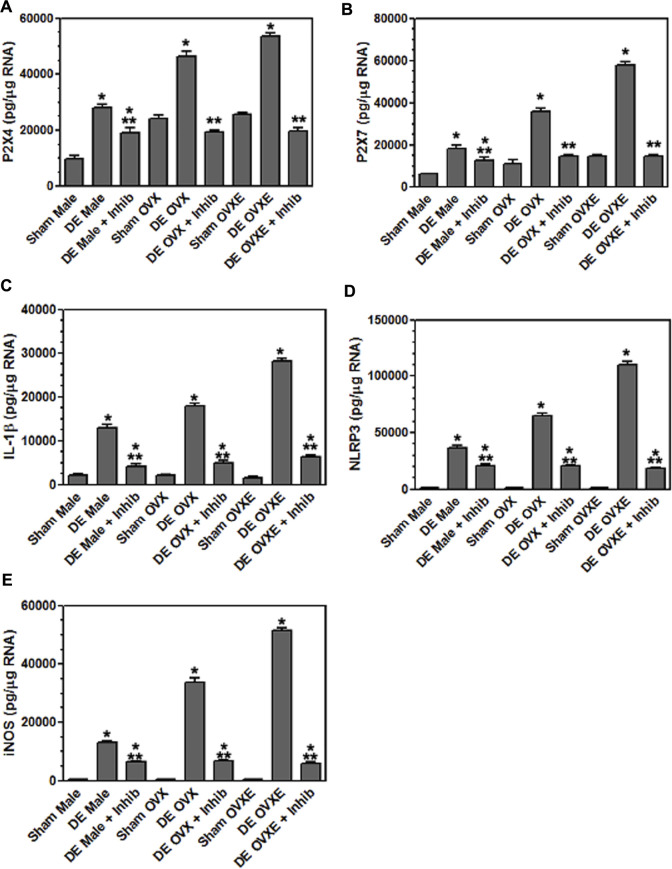
Inhibition of P2X7 reduces inflammatory cytokine expression in microglia. Male, OVX, and OVXE rats had the left exorbital gland removed and were administered P2X7 inhibitor (A804598, 10 mg/kg, sc X 4 days) or untreated. At 14 days post-surgery, rats were perfused with PBS and microglia were isolated and analyzed as in Figure 4. **(A)** P2X4, **(B)** P2X7, **(C)** IL-1*β*, **(D)** NLRP3 and **(E)** iNOS. **p < 0.01 versus sham group. *N* = 8 rats per group. Data presented as mean ± SEM.

**FIGURE 10:**
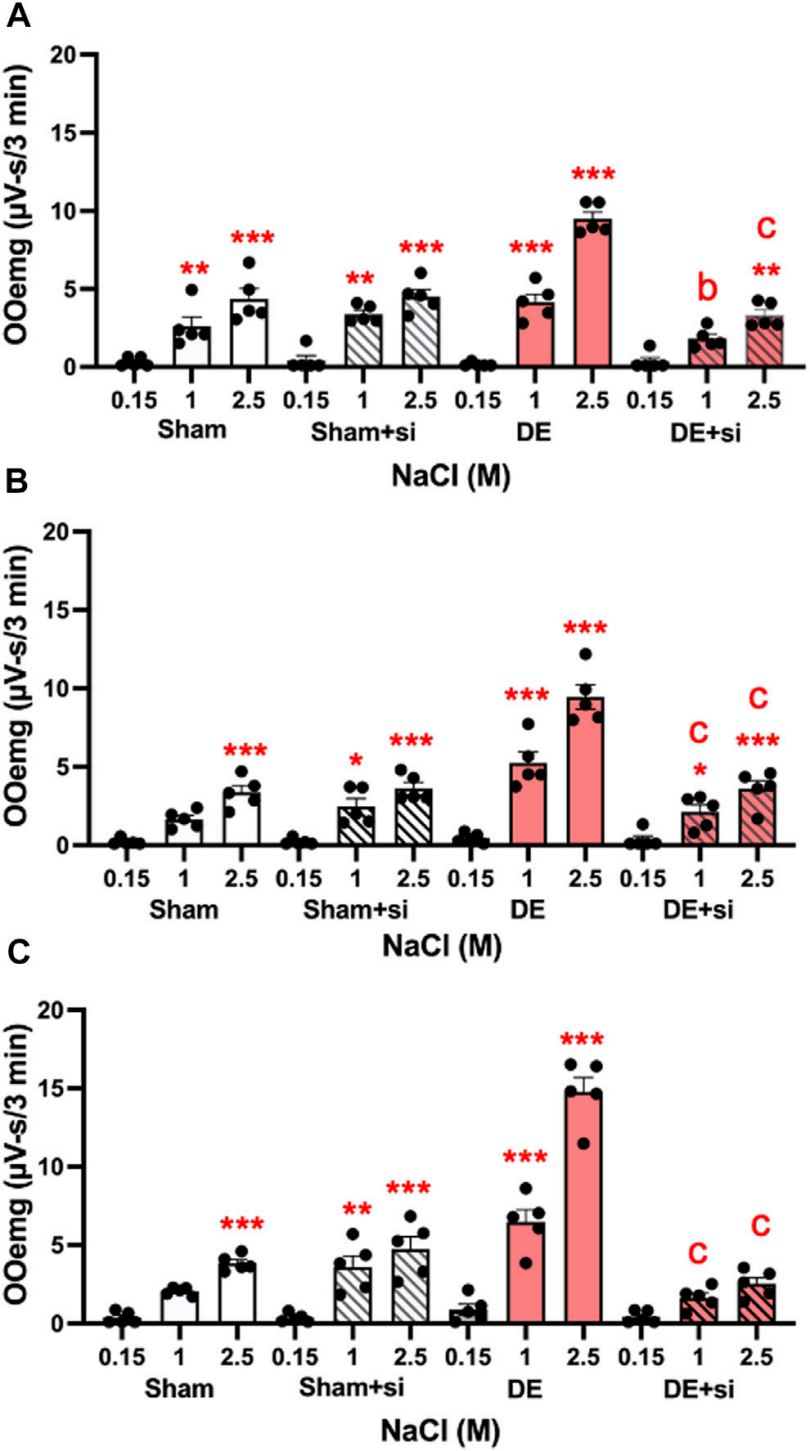
Knockdown of peripheral P2X7R by siRNA injection into TG reduces HS-evoked OOemg in DED rats. siRNA for P2X7R inhibits TMJ-evoked OOemg activity in: **(A)** male, **(B)** OvX and **(C)** OvXE females 3 days prior to recording. Note that responses to HS-evoked OOemg responses in sham rats were not affected. Open bars = sham; open shaded bars = sham + siRNA: Open red bars = 14 days DED; open red shaded bars = 14-day DED + siRNA. *p < 0.05, **p < 0.01 versus 0.15 M NaCl stimulation; *a* = *p*< 0.05, *b* = *p*< 0.01 siRNA treated versus untreated rats. *N* = 5 rats per group. Data presented as mean ± SEM.

**FIGURE 11:**
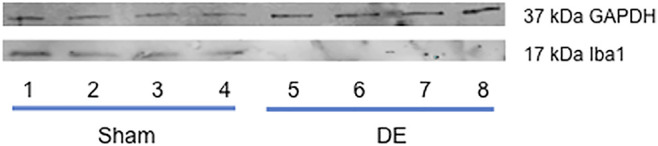
Knockdown of peripheral P2X7R in TG by TG reduces Iba-1 protein in brainstem of DED rats. Example of western blot for total protein for Iba1 in OvX rats based on 20 µg loading samples. Lanes 1-4 = sham; lanes 5-8 = 14-day DED rats.

Figure 1 Flow cytometry data were collected from three rats per group. The percentages in the quadrants were based on total mononuclear cells isolated from the trigeminal brainstem using flow cytometry software by FCS Express. Figures 2, 3, 5. Densitometry data was calculated from four sections per rat (4 rats per group) and expressed as percent positive area. Sections were analyzed without prior knowledge of treatment. Values were compared by one-way analysis of variance (ANOVA) corrected for repeated measures (GraphPad Prism v. 9) on one factor and individual group differences assessed by Tukeys or by Neuman-Keuls. The data were presented as mean ± SEM. Figures 4, 6, 9. Microglia were isolated from the Vsp (8 rats per group). Significant differences were determined by one-way ANOVA and Bonferroni’s multiple comparison test based on values from sham rats of the corresponding treatment group. The data were presented as mean ± SEM. Figures 7, 8, 10. Total OOemg activity was assessed by two-way ANOVA (GraphPad Prism v. 9) and corrected for repeated measures on one factor. Significant treatment effects were assessed by Tukey’s or Newman-Keuls after ANOVA. The data were presented as mean ± SEM and the significant level set at *p* < 0.05, *n* = 5-6 rats per group. Sample size was based on results from previous studies (Rahman et al., 2017; Bereiter et al., 2018), which we calculated would provide 80% power at *p* < 0.05. Three female rats were excluded from further analysis due to low blood pressure at the time of recording. The experiments used sham and DED rats and selected in random order. Figure 11. Protein levels for Iba1 were quantified in Vsp samples from OvX rats by densitometry (4 rats per treatment group) and analyzed by ANOVA.

## Results

### Tear Volume After Exorbital Gland Removal

Male, OvX and OvXE rats displayed no signs of ocular hyperemia or inflammation and gained weight normally over the 14 days after exorbital gland excision. Although fluorescein staining was not performed here, others have used very similar methods for exorbital gland excision and reported no significant change in staining for at least 4 weeks (Meng et al., 2015). Resting tear volume was measured in 90 rats (sham, *n* = 45; DED, *n* = 45). At 14 days after surgery, the tear volume across all DED groups averaged 8.6 ± 0.2 mm/2 min (mean ± SEM) ipsilateral to gland removal and 19.3 ± 0.2 mm/2 min (mean ± SEM) in the contralateral eye (F_1,88_ = 423, *p* < 0.001). In sham rats, tear volume averaged 18.9 ± 0.2 mm/2 min from the left and right eyes. There were no significant sex differences in tear volume for sham or DED groups (*p* > 0.1).

### Microglia Activation in the Trigeminal Brainstem During DED

To determine whether DED was associated with an infiltration of immune cells into the trigeminal brainstem, the spinal trigeminal nucleus (Vsp), which included subnucleus oralis (Vo), interpolaris (Vi) and caudalis (Vc), was dissected from male, OvX, and OvXE rats at 2 days or 14 days post-surgery or following sham surgery. After dissociation the mononuclear cells were isolated and then incubated with fluorescently labeled antibodies to CD11b and CD45 prior to analysis by flow cytometry. Peripheral immune cells express high levels of CD45 while microglia express intermediate levels of CD45. CD11b is present on peripheral monocytes/macrophage and microglia. No infiltrating immune cells (CD45 high cells) were seen in samples from sham groups based on CD11b^+^ and CD45 intermediate expression. Similarly, samples collected at 2 days or 14 days from DED male, OvX, and OvXE rats also had only resident microglia in the brainstem and no evidence of infiltrating immune cells (Figure 1).

Immunohistochemistry (IHC) and densitometry was used to determine if resident microglia in the Vsp were activated in DED rats. The Vsp was removed from sham and 14 days DED male, OvX and OvXE rats. The tissue was fixed, sectioned, stained with anti-Iba-1 and analyzed by confocal microscopy and densitometry for activation of microglia (Figure 2). Overall treatment effects indicated differences between sham and DED rats (F_5,22_ = 3.11, *p* < 0.05). Individual comparisons revealed that microglia at Vo, Vi and Vc levels of Vsp from OvXE rats had a higher expression of Iba1 at 14 days post-surgery compared to OvXE sham rats (*p* < 0.05), while only marginal changes were seen in OvX and males at 14 days post-surgery (Figure 2G). These results suggested that high estrogen status and loss of tear volume interact to enhance microglia activation in the Vsp.

Purinergic P2X7 receptors (P2X7R) are primarily expressed by microglia, the predominant immune competent cell in the CNS, and bind ATP released by injured or stressed neurons to promote expression of cytokines and effector molecules. The effect of DED on P2X7R expression in the Vsp was determined by IHC and densitometry of light microscopy-stained sections (Figure 3G). Note that the fluorescent-stained sections in Figure 3 represent examples only. Overall treatment effects indicated a significant difference for P2X7R between sham and DED rats (F_2,22_ = 3.25, *p* < 0.025). Individual comparisons revealed that P2X7R at Vo and Vc from OvXE rats had a higher expression at 14 days post-surgery compared to OvXE sham rats (*p* < 0.05), while P2X7R expression was not significantly different between OvXE and either OvX or males at 14 days post-surgery (Figure 3G). The expression levels of P2X7R in Vsp subnuclei of OvX and male DED rats were not significantly different from the corresponding sham groups. To determine whether gland removal specifically affected microglia expression of purinergic receptor subtypes, microglia were isolated from whole Vsp from male, OvX, and OvXE rats at 2 and 14 days post-surgery and from sham controls by cell sorting and then analyzed for the expression of P2X7R and P2X4R by real-time PCR (Figure 4). The expression of P2X4R was increased in 2 days and 14 days DED rats in all the groups with the highest level seen under high estrogen conditions (OvXE) (Figure 4A). Similarly, P2X7R expression was increased at 2 days in all DED groups and at 14 days post-surgery in OvX and OvXE, with OvXE rats displaying the highest level of expression (Figure 4B). Overall, microglia displayed increased expression of the purinergic receptors P2X4 and P2X7 at 2 days post-surgery and remained elevated in 14 days DED OvX and OvXE rats. Notably, P2X7R expression was highest in the OvXE group at both 2 and 14 days after gland removal.

Microglia become activated to express cytokines and effector molecules in response to stimuli from their environment. P2X7R is critical for nucleotide-binding domain-like receptor 3 (NLRP3) inflammasome assembly and subsequently for mature IL-1*β* release. To determine the effect of reduced tear volume on NLPR3 expression Vsp tissue sections from the Vo, Vi, and Vc regions were examined by immunohistochemistry (Figure 5). Overall treatment effects indicated differences between sham and DED rats (F_5,21_ = 3.72, *p* < 0.025). Individual comparisons revealed that microglia at Vo and Vc levels of Vsp from OvXE rats had a higher expression of NLRP3 at 14 days post-surgery compared to sham rats (*p* < 0.05), while NLRP3 was only marginally increased in OvX and males at 14 days post-surgery (Figure 5G). To determine whether microglia activated during DED express pro-inflammatory cytokines and effector molecules, which may promote neuroinflammation associated with pain, microglia were isolated from the trigeminal brainstem at 2 and 14 days post-surgery. Microglia were analyzed by real-time PCR for the expression of the pro-inflammatory cytokines, IL-1*β* and TNF*α*; inducible nitric oxide (iNOS) and brain-derived neurotrophic factor (BDNF); NLRP3 inflammasome; and Toll-like receptor 4 (TLR4) (Figure 6). An increase in NLRP3 expression by isolated microglia was seen at 2- and 14-day post-surgery in all groups, while the OvXE group had the highest level of expression (Figure 6A). Microglia displayed increased expression of IL-1*β* at 2 and 14 days DED in male, OVX, and OVXE groups, with greatest increase in expression in OvXE rats (Figure 6B). Microglia displayed a marked increase in the expression of effector molecule, iNOS, at 2 and 14 days DED in all the groups, with the highest increase observed in microglia from OvXE rats (Figure 6C). Toll-like receptor 4 (TLR4) expression on microglia was increased at 2 days DED and at 14 days DED with the highest expression in OvXE rats (Figure 6D). Although microglia also displayed significant increases the expression of tumor necrosis factor alpha (TNF*α*) and BDNF during DED, the overall expression levels were low (Figures 6E,F). The increase in NLRP3 expression was associated with the increased expression of IL-1*β* during DED. Densitometry analyses for GFAP, a marker for astrocytes, revealed no change in expression in DED rats (range = 3.95–5.33% area) compared to sham animals (range = 2.55–4.71% area) (F_5,22_ = 1.49, *p* > 0.1). Overall, these results revealed that microglia became activated in the trigeminal brainstem during DED to express cytokines and effector molecules which promote neuroinflammation.

### Inhibition of Microglia or P2X7R Activation Reduces OOemg Activity

Three approaches were used to determine if inhibiting the activation of microglia or P2X7R affected ocular hyperalgesia as seen by changes in orbicularis oculi muscle electromyography (OOemg) evoked by HS. *Systemic minocycline.* In the first approach, the non-selective glial cell inhibitor, minocycline, was administered systemically daily for 4 days (40 mg/kg) prior to recording and OOemg activity was evoked by instillation of hypertonic saline (HS) in sham and 14 days DED rats. As seen in Figure 7A, males displayed significant increases in HS-evoked OOemg under sham (F_2,38_ = 14.7, *p* < 0.001) and DED conditions (F_2,38_ = 94.7, *p* < 0.001). Minocycline did not affect the OOemg responses in sham males (F_1,9_ = 0.15, *p* > 0.1), whereas evoked responses in DED males were greatly reduced (F_1,10_ = 14.44, *p* < 0.001). As seen in Figure 7B, OvX females also displayed significant increases in HS-evoked OOemg under sham (F_2,36_ = 14.8, *p* < 0.001) and DED conditions F_2,36_ = 121, *p* < 0.001). Minocycline did not affect the OOemg responses in sham OvX females (F_1,9_ = 3.2, *p* < 0.1), whereas the responses in DED OvX females were greatly reduced (F_1,9_ = 14.75, *p* < 0.001). Similarly, OvXE females displayed significant increases in HS-evoked OOemg under sham (F_2,36_ = 12.1, *p* < 0.001) and DED conditions (F_2,36_ = 205, *p* < 0.001, Figure 7C). Unexpectedly, minocycline treatment caused a small increase in HS-evoked OOemg responses in sham OvXE females compared to untreated OvXE females (F_1,9_ = 12.6, *p* < 0.01); however, OOemg responses in DED OvXE females were greatly reduced (F_1,9_ = 42.18, *p* < 0.001), although responses remained elevated compared to untreated OvXE females. These data indicated that minocycline significantly reduced HS-evoked OOemg activity in males as well as in females under low and high estrogen conditions in DED rats.

*Local microinjection of a specific antagonist for P2X7R.* The specific antagonist for P2X7R, A804598 (10 μM, 0.2 µL), was injected into the Vc ipsilateral to exorbital gland excision. As seen in Figure 8A, A804598 markedly reduced HS-evoked OOemg in males (F_7,72_ = 16.97, *p* < 0.001), whereas responses in sham animals were not affected. As seen in Figure 8B, HS-evoked OOemg was markedly reduced in OvX females (F_7,72_ = 27.38, *p* < 0.001) and the reduction in evoked OOemg after Vc injection of A804598 was restricted to DED animals, while responses in sham animals were not affected. Similarly, HS-evoked OOemg was markedly reduced in OvXE DED rats (F_7,72_ = 50.73, *p* < 0.001), whereas the evoked responses in sham animals was not affected (Figure 8C). The magnitude of the drug-induced inhibition of evoked OOemg was significantly greater for OvXE DED animals compared to that seen for male or OvX females (F_5,180_ = 34.4, *p* < 0.001).

To determine whether A804598 would reduce microglia activation in DED or in sham male, OvX, and OvXE rats were administered A804598 (10 mg/kg/day × 4 days, sc) and at 14 days post-surgery, microglia were isolated from the trigeminal brainstem. Isolated microglia were analyzed for the expression of purinergic receptors, P2X4R and P2X7R, IL-1*β*, NLRP3, and iNOS, by real-time PCR (Figure 9). A804598 significantly reduced the expression of P2X4R and P2X7R by isolated microglia in all DED groups. The expression of P2X4R and P2X7R in OvX and OvXE DED groups was reduced to sham levels, whereas male DED rats displayed smaller reductions compared to sham males. The P2X7R antagonist also decreased the expression of IL-1*β*, NLRP3, and iNOS by microglia in male, OvX, and OvXE DED rats. These results demonstrated that inhibition of the P2X7R reduced the activation of microglia in male, OvX, and OvXE DED rats.

*Transcriptional inhibition of P2X7R in TG reduces HS-evoked OOemg activity.* The third approach addressed the role of peripheral P2X7R in ocular hyperalgesia. In the TG P2X7R is expressed mainly by satellite glia that surround neurons. To determine the effect of local peripheral inhibition of P2X7R, siRNA for P2X7R was microinjected into the left TG and 3 days later (i.e., 11 days after surgery) HS-evoked OOemg activity was recorded. As seen in Figure 10A, siRNA for P2X7R caused a significant reduction in HS-evoked OOemg in male DED rats, while responses in sham males were not affected (F_3,16_ = 20.39, *p* < 0.001). Similarly, siRNA injection in OvX females caused a significant reduction in HS-evoked OOemg in DED rats, whereas responses in sham OvX females were not different from non-injected rats (F_3,16_ = 20.38, *p* < 0.001, Figure 10B). As seen in Figure 10C, there was a marked reduction in HS-evoked OOemg responses in OvXE DED rats after siRNA injection, whereas responses in sham OvXE rats were similar to non-injected sham OvXE rats (F_3,16_ = 44.38, *p* < 0.001). Note also that siRNA injection in all DED groups significantly reduced the OOemg responses to 1 M NaCl, an osmolar concentration similar to that found at the center of the ocular surface in severe DED patients (Liu et al., 2009). Knockdown of P2X7R was confirmed by western blot analyses. Intra-TG injection of siRNA markedly reduced P2X7R protein levels in the TG of DED males compared to vehicle injected DED males (0.137 ± 0.003 versus 1.589 ± 0.245 relative intensity, F_1,6_ = 43.2, *p* < 0.001). Similarly, TG injection of siRNA in DED OvXE females was greatly reduced compared to vehicle injected OvXE females (0.009 ± 0.001 versus 0.213 ± 0.027 relative intensity) (F_1,6_ = 53.43, *p* < 0.0001). To determine the effect of intra-TG injection of siRNA for P2X7R on microglia activation in trigeminal brainstem, Vsp tissues were collected from OvX females for western blot analyses of Iba-1 (Figure 11). These results demonstrated that intra-TG injection of siRNA for P2X7R in DED rats caused a marked reduction in protein levels of Iba-1 compared to vehicle injected DED OvX females (0.098 ± 0.003 versus 0.605 ± 0.053, mean ± SEM, relative intensity) (F_1,6_ = 89.14, *p* < 0.0001).

## Discussion

The present study used a rat model for aqueous deficient DED to address two unresolved issues concerning ocular hyperalgesia and the contribution of neuroimmune responses. First, does activation of P2X7R in trigeminal pain pathways play a role in ocular hyperalgesia and immune cell activation? Secondly, does estrogen status play a significant role in ocular hyperalgesia and immune cell activation? These results revealed that activation of P2X7R in the TG and Vsp contributed to enhanced ocular surface-evoked nociceptive behavior and neuroimmune function in DED rats. Estrogen treatment further enhanced ocular hyperalgesia and microglia activation in a P2X7R-dependent manner in female DED rats.

Tear hyperosmolarity and loss of tear film integrity are diagnostic features of DED (Baudouin et al., 2013; Craig et al., 2017; Willcox et al., 2017). Exorbital gland excision is a valid model for aqueous deficient DED which results in a ∼40% reduction in tear volume (Fujihara et al., 2001; Meng et al., 2015; Rahman et al., 2015; Mecum et al., 2019) and elevates levels of pro-inflammatory molecules in the anterior eye segment (Joossen et al., 2016), consistent with clinical signs seen in DED patients (Na et al., 2012; Nicolle et al., 2018). A challenge for preclinical studies of ocular pain is the ability to measure behaviors that can be reasonably interpreted as eye pain in humans. Several methods have been used to estimate chronic ocular hyperalgesia in animals: spontaneous and evoked eyeblink rates, palpebral opening and forelimb eye wiping to ocular surface stimulation (de Castro et al., 1998; Bates et al., 2010; Yorek et al., 2016; Fakih et al., 2019; Mecum et al., 2020). Recently, we reported that HS-evoked OOemg activity was a reliable surrogate for ocular hyperalgesia in anesthetized male rats (Rahman et al., 2017). HS-evoked long duration, squint-like OOemg activity that was enhanced in DED rats, corresponded well to cornea-evoked eye wiping behavior and involved TRPV1 activation, results that were consistent with the notion of HS-evoked OOemg as a nociceptive behavior (Bereiter et al., 2018).

The initiation and maintenance of nociceptive behavior in preclinical studies of DED likely involves peripheral and central neural mechanisms. In the periphery hyperosmolar stress induces an increase in ATP levels in the tears of DED patients which is the preferred nucleotide for activation of P2X7R (Guzman-Aranguez et al., 2017). P2X7R is closely linked to inflammation and is necessary for NLRP3 inflammasome assembly and the production and release of proinflammatory cytokines such as IL-1*β* (Di Virgilio et al., 2017). P2X7R is expressed by satellite glia that surround TG neurons and mediates crosstalk between neurons and non-neural cells in the TG (Belzer et al., 2011; Nowodworska et al., 2017). The present study found that microinjection of siRNA for P2X7R into the TG markedly reduced HS-evoked OOemg activity and Iba-1 expression by microglia in trigeminal brainstem of DED rats. P2X7R also is expressed by corneal and conjunctival epithelial cells (Minns et al., 2016) and NLRP3 suggesting that increased levels of proinflammatory cytokines derive from multiple sources in DED (Zheng et al., 2014). P2X7R also is expressed by goblet cells and is critical for mucin secretion suggesting a role in mechanical transduction by corneal nociceptors (Puro 2021). Loss of lubrication at the ocular surface leads to increased friction during lid wiping and subsequent enhanced corneal nociceptor activation during periods of increased blinking (van Setten 2020). These data suggest that loss of tear film integrity induces P2X7R activation in the eye and TG which, through increased secretion of proinflammatory molecules, contributes to ocular symptoms and neuroimmune responses in DED. It noteworthy that P2X7R requires a higher concentration of ATP for activation and displays limited desensitization compared to other purinergic receptor subtypes suggesting the contribution to DED symptoms and ocular surface homeostasis may be greatest under conditions of moderate to severe inflammation.

Microglia are resident immune cells in the CNS and play a critical role in maintenance of chronic pain (Chen et al., 2018; Inoue and Tsuda 2018). Although microglia display rapid and persistent activation following even brief periods of nociceptor activity (Hathway et al., 2009; Gruber-Schoffnegger et al., 2013), the mechanisms for microglia involvement in chronic pain are not completely known. We confirmed that the expression of P2X7R and Iba-1 derived from resident microglia and not from infiltrating monocytes by flow cytometry and cell sorting followed by qRT-PCR analyses. These results agreed with previous preclinical studies of neuropathic and inflammatory pain in which spinal cord samples were analyzed flow cytometry and found no evidence of monocyte infiltration (Denk et al., 2016; Lopes et al., 2017; Fernandez-Zafra et al., 2019). In the present study, isolated microglia also expressed elevated levels of P2X4R in Vsp tissue samples at 2 and 14 days after exorbital gland removal. Although P2X7 and P2X4 do not normally form heterotrimers, immunoprecipitation studies indicate interaction of purinergic subtypes may act to influence immune cell function (Boumechache et al., 2009; Sakaki et al., 2013; Perez-Flores et al., 2015). Thus, it cannot be excluded that P2X4 as well as P2X7 may have contributed to the increase ocular hyperalgesia and expression of proinflammatory cytokines in this model of DED. Given the differences in threshold concentrations of nucleotides necessary to activate P2X4 and P2X7, microglia that express both subtypes could be expected to respond to a greater range of concentrations and patterns of molecular signals following nociceptor stimulation (Kato et al., 2016). P2X7R activation in microglia induces multiple downstream signaling pathways and is sufficient to cause long-term potentiation of dorsal horn neurons (Chu et al., 2010; Kronschlager et al., 2016). iNOS expression also was markedly increased in isolated microglia from Vsp tissue samples which was consistent with the involvement of glutamatergic pathways (Freire et al., 2009; Gruber-Schoffnegger et al., 2013).

The second goal of this study was to determine whether estrogen status played a significant role in ocular hyperalgesia and neuroinflammation in DED. These results strongly indicated that acute estrogen treatment in ovariectomized female rats enhanced HS-evoked OOemg activity and the expression of P2X7R in the Vsp. Densitometry of Vsp tissue sections revealed that Iba-1, NLRP3 and P2X7R positive areas were greater in Vc of OvXE DED groups than in sham OvXE rats. The Vc receives a significant direct input from TG neurons that supply the ocular surface (Marfurt and del Toro 1987; Marfurt and Echtenkamp 1988; Panneton et al., 2010). The Vc also displayed a high number of estrogen receptor positive neurons compared to other portions of the Vsp (Bereiter et al., 2005; VanderHorst et al., 2005). Estrogen treatment in DED rats induced significantly higher levels of expression by isolated microglia from whole Vsp samples for P2X7R, P2x4R, NLRP3, IL-1*β*, TLR4 and iNOS compared to sham OvXE rats or to OvX or male animals. This suggested an interaction between estrogen treatment and loss of tear volume which increased the level of inflammatory molecules compared to either treatment alone. P2X7, together with P2X4 and Toll-like receptors (TLRs), mediates assembly of the NLRP3 inflammasome which leads to activation of caspase-1 and cleavage of pro-IL-1*β* into mature IL-1*β*. Although TNF*α* and BDNF expression also were increased in isolated microglia of DED rats, these expression levels were much lower than for other pro-inflammatory molecules. To determine if P2X7R activation in the Vsp altered ocular hyperalgesia in an estrogen-dependent manner, the selective P2X7R antagonist, A804598, was microinjected into the Vc in DED and sham rats. These results revealed a significant reduction in HS-evoked OOemg in all DED groups and only minor changes in sham animals. To determine whether the A804598-induced reduction in HS-evoked OOemg activity in DED rats depended on P2X7R, the drug was administered systemically for 4 days prior to tissue collection. Isolated microglia displayed significant reductions in P2X7R, P2x4R, NLRP3, IL-1*β* and iNOS in all DED groups. These data suggested that P2X7R activation was necessary for enhanced ocular hyperalgesia in DED.

Several studies have reported that intrathecal injection of minocycline reduced mechanical hyperalgesia in males but not female rodents in nerve injury and arthritic pain models (Sorge et al., 2015; Mapplebeck et al., 2018; Fernandez-Zafra et al., 2019). By contrast, we found that systemic minocycline reduced HS-evoked OOemg activity of DED rats of both sexes. Several methodological differences may have contributed to this discrepancy. First, our DED model did not involve direct nerve injury, but rather resulted in changes in tear film integrity and corneal sensitivity that developed over several days (Meng et al., 2015). Second, previous studies applied minocycline as a single intrathecal injection, while we administered minocycline systemically in four daily doses. Interestingly, in a model for bone cancer which also develops slowly, intrathecal administration of minocycline effectively reduced mechanical hyperalgesia in female rats (Yang et al., 2015). Lastly, we cannot exclude that estrogen treatment given as a single injection to OvX female rats the day before OOemg recording or tissue collection may have led to results that may have differed from those seen in normal cycling female rats.

These data indicated that P2X7R activation in peripheral and central sites in the trigeminal pain pathway was a significant factor in mediating enhanced ocular hyperalgesia and neuroinflammation in this model for aqueous deficient DED. Estrogen status likely played a significant role in mediating the magnitude of evoked OOemg activity and markers for neuroinflammation in DED.

## Data Availability

The raw data supporting the conclusion of this article will be made available by the authors, without undue reservation.
